# Supplementary material for: Karyotype Evolution in 10 Pinniped Species: Variability of Heterochromatin versus High Conservatism of Euchromatin as Revealed by Comparative Molecular Cytogenetics
Source: Genes (Basel). 2020 Dec 10;11(12):1485. doi: 10.3390/genes11121485 (PMC7763226; doi:10.3390/genes11121485)
Supplement: Supplementary file 1 [file genes-11-01485-s001.zip › Suppl Mat-Pinnipeds/Supplementary Figure S1.doc]

Figure S1. GTG-banded karyotype of: a) the Steller sea lion (EJUB, 2n=36), b) the walrus (OROS, 2n=32), with the assignment of homology to human (HSA), dog (CFA) and stone marten (MFO) chromosomes. Nucleolus organizer regions are marked as NOR. The square denotes the centromere position on the corresponding chromosome. * — heterochromatin regions not painted by any dog probe. The dog chromosomal nomenclature follows Yang et al.[1].

References

1.	Yang, F.; O’Brien, P.C.; Milne, B.S.; Graphodatsky, A.S.; Solanky, N.; Trifonov, V.; Rens, W.; Sargan, D.; Ferguson-Smith, M.A. A complete comparative chromosome map for the dog, red fox, and human and its integration with canine genetic maps. Genomics 1999, 62, 189–202.
