# Supplementary material for: Karyotype Evolution in 10 Pinniped Species: Variability of Heterochromatin versus High Conservatism of Euchromatin as Revealed by Comparative Molecular Cytogenetics
Source: Genes (Basel). 2020 Dec 10;11(12):1485. doi: 10.3390/genes11121485 (PMC7763226; doi:10.3390/genes11121485)
Supplement: Supplementary file 1 [file genes-11-01485-s001.zip › Suppl Mat-Pinnipeds/Supplementary Material 1.doc]

### Supplementary Material 1

### *Nucleolus Organizer Regions in Seals and Walrus*

Nucleolus Organizer Regions (NORs) contain tandem arrays of ribosomal genes (rDNA) [[1]](https://paperpile.com/c/PNDgIW/BZfw). The number, size and location of the NORs may vary in mammalian orders. In different species of canoid carnivores with varying level of genome rearrangements from one to six sites of ribosomal genes were described on autosomes.

In pinnipeds the single NOR site is remarkably conserved. It was previously shown using the Ag-staining which reveals active NOR sites [[2]](https://paperpile.com/c/PNDgIW/ZfHm). FISH with the 5.8S, 18S, 28S rDNA probe identifies all ribosomal sites regardless of their transcriptional activity. By FISH we show the presence of the main site on the chromosome homologous to ACK15 (MFO 15) in the genomes of walrus and all studied seals. Interestingly, two small additional sites were identified here in the karyotypes of the common seal (PVIT 6p (m3)) and ringed seal (PHIS Yp).

The NOR localization on the Y-chromosome is not common for mammals. In Carnivora NORs are present on the Y-chromosome only in Canidae (wolf, dog, and raccoon dogs)[[3–11]](https://paperpile.com/c/PNDgIW/GQbD+vPnW+jfkU+iI7X+Wzyv+OqoV+bp19+OR6H+9HzW). An additional NOR site on Yp in ringed seal (PHIS Yp) was revealed here in a single individual and it leaves open the question whether it is individual, polymorphic or characteristic for entire species, especially because a subtelocentric Y has been previously reported for this species[[12]](https://paperpile.com/c/PNDgIW/I1gvs) unlike metacentric Y found here.

It was noted that the unique NOR is usually located in the interstitial position whereas multiplicity of NOR sites correlates with location at the distal part or on the short arms of acrocentric chromosomes[[13,14]](https://paperpile.com/c/PNDgIW/Qc8gs+28KpM). NORs are frequently involved in exchanges, and their location on the chromosome ends would not lead to chromosome imbalances after meiosis. The location of the additional autosomal site of the rDNA in the harbor seal violates this pattern. Interstitial position of the NOR on the PVIT 6p does not coincide with any of the sites identified in other representatives of the Carnivores. Further studies needed to determine if this de novo NOR site contains working rDNA genes.

References

1.	[McStay, B. Nucleolar organizer regions: genomic “dark matter” requiring illumination. *Genes Dev.* **2016**, *30*, 1598–1610.](http://paperpile.com/b/PNDgIW/BZfw)

2.	[Arnason, U. Localization of nucleolar organizing regions in pinniped karyotypes. *Hereditas* **1981**, *94*, 29–34.](http://paperpile.com/b/PNDgIW/ZfHm)

3.	[Mäkinen, A.; Zijlstra, C.; de Haan, N.A.; Mellink, C.H.; Bosma, A.A. Localization of 18S + 28S and 5S ribosomal RNA genes in the dog by fluorescence in situ hybridization. *Cytogenet. Cell Genet.* **1997**, *78*, 231–235.](http://paperpile.com/b/PNDgIW/GQbD)

4.	[Mäkinen, A. The standard karyotype of the silver for (Vulpes fulvus Desm.). Committee for the standard karyotype of Vulpes fulvus Desm. *Hereditas* **1985**, *103*, 171–176.](http://paperpile.com/b/PNDgIW/vPnW)

5.	[Mäkinen, A. The standard karyotype of the blue fox (Alopex lagopus L.): Committee for the standard karyotype of Alopex lagopus L. *Hereditas* 2008, *103*, 33–38.](http://paperpile.com/b/PNDgIW/jfkU)

6.	[Mäkinen, A.; Kuokkanen, M.T.; Valtonen, M. A chromosome-banding study in the Finnish and the Japanese raccoon dog. *Hereditas* **1986**, *105*, 97–105.](http://paperpile.com/b/PNDgIW/iI7X)

7.	[Cavagna, P.; Menotti, A.; Stanyon, R. Genomic homology of the domestic ferret with cats and humans. *Mamm. Genome* **2000**, *11*, 866–870.](http://paperpile.com/b/PNDgIW/Wzyv)

8.	[Pathak, S.; Van Tuinen, P.; Merry, D.E. Heterochromatin, synaptonemal complex, and NOR activity in the somatic and germ cells of a male domestic dog, Canis familiaris (Mammalia, Canidae). *Cytogenet. Cell Genet.* **1982**, *34*, 112–118.](http://paperpile.com/b/PNDgIW/OqoV)

9.	[Kopp, E.; Mayr, B.; Schleger, W. Nucleolus organizer regions on chromosomes of the domestic dog. *J. Hered.* **1982**, *73*, 73.](http://paperpile.com/b/PNDgIW/bp19)

10.	[Mayr, B.; Geber, G.; Auer, H.; Kalat, M.; Schleger, W. Heterochromatin composition and nucleolus organizer activity in four canid species. *Can. J. Genet. Cytol.* **1986**, *28*, 744–753.](http://paperpile.com/b/PNDgIW/OR6H)

11.	[Mandahl, N.; Fredga, K. A comparative chromosome study by means of G-, C-, and NOR-bandings of the weasel, the pygmy weasel and the stoat (Mustela, Carnivora, Mammalia). *Hereditas* **1980**, *93*, 75–83.](http://paperpile.com/b/PNDgIW/9HzW)

12.	[Árnason, Ú. Comparative chromosome studies in Pinnipedia. *Hereditas* **1974**, *76*, 179–225.](http://paperpile.com/b/PNDgIW/I1gvs)

13.	[Gerbault-Seureau, M.; Cacheux, L.; Dutrillaux, B. The Relationship between the (In-)Stability of NORs and Their Chromosomal Location: The Example of Cercopithecidae and a Short Review of Other Primates. *Cytogenet. Genome Res.* **2017**, *153*, 138–146.](http://paperpile.com/b/PNDgIW/Qc8gs)

14.     [Dutrillaux, A.-M.; Carton, B.; Cacheux, L.; Dutrillaux, B. Interstitial NORs, Fragile Sites, and Chromosome Evolution: A Not So Simple Relationship - The Example of ***Melolontha melolontha*** and Genus ***Protaetia*** (Coleoptera: Scarabaeidae). *Cytogenetic and Genome Research* 2016, *149*, 304–311.](http://paperpile.com/b/PNDgIW/28KpM)
