# Supplementary material for: Karyotype Evolution in 10 Pinniped Species: Variability of Heterochromatin versus High Conservatism of Euchromatin as Revealed by Comparative Molecular Cytogenetics
Source: Genes (Basel). 2020 Dec 10;11(12):1485. doi: 10.3390/genes11121485 (PMC7763226; doi:10.3390/genes11121485)
Supplement: Supplementary file 1 [file genes-11-01485-s001.zip › Suppl Mat-Pinnipeds/Supplementary Material 2.doc]

**Supplementary Material 2**

*The Sex Chromosomes Features in Pinniped Karyotypes*

The conservatism of the X-chromosome was described by Ohno et al [[1]](https://paperpile.com/c/PNDgIW/vIzC2) for eutherian mammals. The length of the “original” type X-chromosome is 5.4% of the female haploid set. Our data for seals and walrus is consistent with literature information for pinnipeds and demonstrates that the X chromosome is a medium-sized submetacentric with conservative GTG-banding pattern similar to that for the “original” type X [[2]](https://paperpile.com/c/PNDgIW/I1gvs). However, a strikingly different X chromosome was described for the ribbon seal (*Histriophoca fasciata*): it has the secondary constriction in the q-arm and a relative length of 8.8% versus 5.5% in all other phocids possibly due to accumulation of heterochromatin [[2]](https://paperpile.com/c/PNDgIW/I1gvs).

The Y chromosome is the smallest element in pinniped karyotypes with exception of an unusually large Y chromosome in the southern elephant seal, comparable in size to Xq [[3]](https://paperpile.com/c/PNDgIW/Xc77L). We show a variety in morphology and nucleotide content of the Y-chromosome in the examined species. Mainly the Y-chromosome is submeta- or subtelocentric. The ringed seal Y-chromosome is almost symmetric metacentric due to an additional cluster of ribosomal genes (Figure 7c). Published data also demonstrates the interspecific morphological diversity of this sex chromosome in different pinnipeds [[2]](https://paperpile.com/c/PNDgIW/I1gvs). So the Y chromosome variability in pinnipeds represents one more example of changes resulting from heterochromatin variation in otherwise conserved pinniped karyotypes. It is hard to fully assess the scope of this variability due to a limited number of individuals of each species studied in pinnipeds.

References

1.	X-autosome ratio and the behavior pattern of individual X-chromosomes in placental mammals. *Chromosoma* **1964**, *15*, 14–30.

2.	[Árnason, Ú. Comparative chromosome studies in Pinnipedia. *Hereditas* **1974**, *76*, 179–225.](http://paperpile.com/b/PNDgIW/I1gvs)

3.	[Robinson, T.J.; Condy, P.R. The chromosomes of the southern elephant seal, Mirounga leonina (Phocidae: Mammalia). *Cytogenet. Cell Genet.* **1979**, *23*, 157–162.](http://paperpile.com/b/PNDgIW/Xc77L)
