# Supplementary material for: Karyotype Evolution in 10 Pinniped Species: Variability of Heterochromatin versus High Conservatism of Euchromatin as Revealed by Comparative Molecular Cytogenetics
Source: Genes (Basel). 2020 Dec 10;11(12):1485. doi: 10.3390/genes11121485 (PMC7763226; doi:10.3390/genes11121485)
Supplement: Supplementary file 1 [file genes-11-01485-s001.zip › Suppl Mat-Pinnipeds/TableS1_Correspondence of pinniped chrom+ACK+.doc]

### Table S1. Correspondence of pinniped (the bearded seal (EBAR), the New Zealand fur seal (AFOR), the northern fur seal (CURS) and the New Zealand sea lion (PHOK)) chromosomes with dog (CFA), human (HSA), Ancestral Carnivore Karyotype (ACK [**[1]**](https://paperpile.com/c/PNDgIW/4sl9+zZTU))^1^, stone marten (MFO [**[2]**](https://paperpile.com/c/PNDgIW/4sl9+zZTU))^2^ and cat (FCA [**[2]**](https://paperpile.com/c/PNDgIW/4sl9+zZTU))^2^ chromosomes. Correspondence with human chromosome is based on our FISH results, human chromosome segments are designated according to published data.[**[1,2]**](https://paperpile.com/c/PNDgIW/4sl9+zZTU)

| EBAR | AFOR | CURS | PHOK | CFA | HSA | ACK | MFO | FCA |
| --- | --- | --- | --- | --- | --- | --- | --- | --- |
| 1 | 1 | 1 | 1 | 20/23/35/30/27 | 19p/3/21 | 1 | 2 | A2p/C2 |
| 4 | 4 | 3 | 2 | 22/28/19/36/33/28 | 13/2q | 3 | 3 | A1p/C1q |
| 2 | 2 | 6 | 3 | 16/28/15/19/32/13/3 | 4q/8p/4p+q | 2 | 1 | B1 |
| 3 | 3 | 2 | 4 | 13/34/17/6/5/15/2/5 | 8q/1p+q | 4 | 4 | F2/C1p |
| 10 | 5 | 4 | 5 | 11/2/3/4/35 | 5 | 5 | 5 | A1q |
| 5pprox+5q | 9 | 8 | 9 | 2/29/10/15/10 | 10p/12/22 | 8 | 9 | B4 |
| 9 | 7 | 9 | 7 | 28/24/10/17 | 20/2p | 9 | 8 | A3 |
| 6 | 6 | 5 | 6 | 3/25/8 | 15/14 | 7 | 7 | B3 |
| 7 | 8 | 7 | 8 | 37/12/1 | 6 | 6 | 6 | B2 |
| 5pdist | 10p+qprox | 10p+qprox | 10p+qprox | 6 | 7/16p | 18 | 18 | E3 |
| 8p | 10qdist | 10qdist | 10qdist | 37/8 | 1q | 16 | 16 | F1 |
| 8q | 12 | 13 | 11 | 4/31 | 10q | 11 | 12 | D2 |
| 11 | 14 | 12 | 12 | 5/21/18 | 11 | 12 | 10 | D1 |
| 12 | 13 | 11 | 13 | 18/14/16 | 7 | 10 | 11 | A2q |
| 13 | 11 | 15 | 15 | 26/7/1 | 12/22/18 | 13 | 13 | D3 |
| 14 | 15 | 14 | 14 | 1/11/9 | 9 | 14 | 14 | D4 |
| 15 | 16 | 16 | 16 | 9/5 | 17 | 15 | 15 | E1 |
| 16 | 17 | 17 | 17 | 1/2/5 | 19q/16q | 17 | 17 | E2 |
| X | X | X | X | X | X | X | X | X |

References

1.	Beklemisheva, V.R.; Perelman, P.L.; Lemskaya, N.A.; Kulemzina, A.I.; Proskuryakova, A.A.; Burkanov, V.N.; Graphodatsky, A.S. The Ancestral Carnivore Karyotype As Substantiated by Comparative Chromosome Painting of Three Pinnipeds, the Walrus, the Steller Sea Lion and the Baikal Seal (Pinnipedia, Carnivora). *PLoS One* **2016**, *11*, e0147647.

2.	Nie, W.; Wang, J.; Su, W.; Wang, D.; Tanomtong, A.; Perelman, P.L.; Graphodatsky, A.S.; Yang, F. Chromosomal rearrangements and karyotype evolution in carnivores revealed by chromosome painting. *Heredity* 2012, *108*, 17–27.
